# Landscape and Health: Connecting Psychology, Aesthetics, and Philosophy through the Concept of *Affordance*

**DOI:** 10.3389/fpsyg.2016.00571

**Published:** 2016-05-03

**Authors:** Laura Menatti, Antonio Casado da Rocha

**Affiliations:** ^1^Faculty of Sciences, Universidad de ChileSantiago, Chile; ^2^IAS-Research Center for Life, Mind, and Society, University of the Basque CountryDonostia-San Sebastián, Spain

**Keywords:** affordance, agency, ecological psychology, health, landscape, naturalistic aesthetics, well-being, perception

## Abstract

In this paper we address a frontier topic in the humanities, namely how the cultural and natural construction that we call landscape affects well-being and health. Following an updated review of evidence-based literature in the fields of medicine, psychology, and architecture, we propose a new theoretical framework called “processual landscape,” which is able to explain both the health-landscape and the medical agency-structure binomial pairs. We provide a twofold analysis of landscape, from both the cultural and naturalist points of view: in order to take into account its relationship with health, the definition of landscape as a cultural product needs to be broadened through naturalization, grounding it in the scientific domain. Landscape cannot be distinguished from the ecological environment. For this reason, we naturalize the idea of landscape through the notion of *affordance* and Gibson’s ecological psychology. In doing so, we stress the role of agency in the theory of perception and the health-landscape relationship. Since it is the result of continuous and co-creational interaction between the cultural agent, the biological agent and the *affordances* offered to the landscape perceiver, the processual landscape is, in our opinion, the most comprehensive framework for explaining the health-landscape relationship. The consequences of our framework are not only theoretical, but ethical also: insofar as health is greatly affected by landscape, this construction represents something more than just part of our heritage or a place to be preserved for the aesthetic pleasure it provides. Rather, we can talk about the right to landscape as something intrinsically linked to the well-being of present and future generations.

## Introduction

It is common to hear that contact with nature, in its many and diverse forms, promotes human health. But how are we to understand this connection? A recent *Frontiers in Psychology* article (Kuo, 2015) identifies several environmental factors, physiological and psychological states, behaviors or conditions, each of which has been empirically tied to nature and has implications for specific physical and mental health outcomes. It might be true that walking through the surrounding landscape, to name just an instance, has a positive or restorative effect on health, but how can we understand the myriad of studies and literature reviews on that topic? And if there is a connection between nature and human health, what is the theoretical framework in which we can understand this hypothesis? To answer these questions, we have analyzed the existing literature on the landscape-health relationship in order to gain a comprehensive idea of the different research areas currently being explored, and to discuss their implications.

There is, in fact, a large body of evidence attesting to the role played by landscape in the treatment, recovery and maintenance of human health. We analyze the majority of the papers and theories that address this relationship from a medical, psychological, and philosophical point of view. However, even though some of the evidence-based papers suggest a theoretical interpretation, they generally lack a comprehensive theory capable of explaining the connection between human beings, landscape and health.

For this reason, we hypothesize that a comprehensive framework for explaining the health-landscape relationship could be established by including the concepts of agency and *affordance* in a new theory of landscape. We therefore propose a new notion of landscape which combines both the cultural and naturalist approaches, and as a result we introduce the idea of *processual landscape* to explain both the health-landscape and the medical agency-structure binomial pairs.

In introducing this new framework, we follow both a naturalistic and cultural methodology. Human beings live embedded in landscape and they perceive it through their whole body; it affects their well-being. The common understanding of landscape today is still the cultural one, which maintains a delicate balance between aesthetics and cultural studies, history and art theory. In this paper we provide a brief history of the concept of landscape from a visual, perceptional, and cultural point of view. However, in order to bridge the gap between social and natural sciences in their approach to this concept, we take a step forward in the naturalization of landscape. In order to understand what landscape is and how it affects our health we have to address biological and ecological theories of perception of the environment.

Our hypothesis is that, thanks to ecological psychology and the consequent theory of *affordance*, it is possible to naturalize landscape by introducing ideas from the scientific domain into a body of literature that has, for decades, remained exclusively cultural. Human beings are an active part of the process of co-creating landscape, both from a cultural and an ecological point of view. Their life, body and perception (which here we ground in the theory of affordance) cannot be detached from landscape. Their mental and physical health depends and relies on it. This agential dimension of the notion of processual landscape, we argue, provides the missing connection with health and well-being.

The paper is structured as follows: in section “Main Theoretical Issues and Approaches” we review the main theoretical issues and approaches to the debate on health and landscape, and in section “Research Focused on Restorative Environments” we analyze the psychological research on restorative environments connected to stress reduction and attention theories. In section “Evidence of the Health-Landscape Relationship: Toward a New Definition of Health” we analyze the medical evidence of the landscape-health relationship, the social determinants of health (SDH) theory, and in doing so we propose a new definition of health connected to agency. In section “What is Landscape? The Cultural Approach and Beyond” we provide a brief history of the cultural concept of landscape from the visual and artistic points of view. In section “A Step Toward Biology and Ecology: Naturalization of Landscape” we broaden the definition of landscape to include biology and ecology. In section “Ecology of Perception: a New Role for the Perceiver” we naturalize the concept of landscape by taking into consideration the role of the perceiver and the *affordances* offered by landscape. In section “Processual Landscape: A Framework Connecting Health and Landscape” we propose the theoretical framework of processual landscape, which is able to explain and ground the health-environment relationship through the twofold idea of cultural and biological agency. We then conclude with a recapitulation and some general remarks on the effectiveness of the framework proposed and its ethical implications.

## Main Theoretical Issues and Approaches

Many papers in contemporary literature analyze data series related to how well-being could be effectively improved by exposure to natural landscapes (see Coles and Millman, 2013). From architecture and the humanities to medicine and ecology, the evidence reported highlights the fact that well-being, health and natural landscape are correlated. Below, we summarize some of the main approaches found in the literature: psychological, philosophical, medical and those based on social determinants. We have divided this section into two parts: the first one addresses research focused on restorative environments and their connection with psychological and philosophical theories of place. These studies, based on stress reduction and attachment to place, were the first to illustrate the correlation between landscape and health during the 1970 and 1980s. In the second section, the analysis of the literature is focused on the concept of health, and how over recent decades health and well-being have been connected to landscape through medical evidence and studies on their social determinants. By presenting these various theories, we acknowledge their pioneering role in the debate while at the same time pointing out that they lack a comprehensive theoretical framework. The aim of this paper is to provide a new, broader framework, which is both cultural and ecological.

### Research Focused on Restorative Environments

Psychology and philosophy stress the importance of the relationship between health and landscape in different ways. Psychology, for instance, addresses this issue through psycho-evolutionary frameworks and the so-called ART theory, all of which focus on stress reduction. Philosophy, on the other hand, proposes a place-attachment approach which originates from phenomenology and human geography. One of the most important theories of place-attachment is called *topophilia*, which has nowadays been expanded to include the concept of *biophilia*. All these theories demonstrate, from different points of view, the intimate connection between our body, our health and the landscape in which we live, or merely look at.

#### Stress Reduction (Theories of Evolution)

Psychophysiological theories of stress reduction (Ulrich, 1983; Ulrich et al., 1991) have shown that being in a non-threatening natural environment reduces the physical indicators connected with stress, such as blood pressure, heart rate, serum cortisol, and skin conductance (see also the studies connecting access to green spaces and health in Ward Thompson et al., 2010, 2014; Ward Thompson, 2011; Curl et al., 2015). In these works, stress is usually defined as the process by which individuals respond psychologically, physiologically, and often behaviorally to situations that challenge or threaten their well-being (Ulrich et al., 1991, p. 202).

These theories played a pioneering role in the determination of health by natural settings, yet can be called into question from a theoretical point of view. Ulrich (1983) and Ulrich et al. (1991), for instance, proposes a psycho-evolutionary framework to explain the role of landscape in reducing stress, based on the following elements: (1) response to natural setting is basically unconscious and related to adaptive responses to nature; (2) nature contact (even a simple view) can rapidly evoke positive stress reduction effects; (3) the origin of the response relies on the survival of the human species. According to this framework, the color green (signifying, for example, refuge) would be less stressful than red or yellow, which signify fire (Ulrich et al., 1991; Ward Thompson, 2011, p. 193). The reason for this preference is directly related to the link between evolution and aesthetic response: nature helped proto-humans to recover from acute stress and to prepare them for the next survival task (see also Hartig et al., 2014, p. 217). The psycho-evolutionary theory has the merit of initiating the debate on the naturalization of landscape (see Appleton, 1975; Dutton, 2006, 2009; Di Summa-Knoop, 2014), yet it poses a number of problems. Firstly, an evolution-based theory of stress reduction or landscape preference cannot be demonstrated. Secondly, it risks relying on *ad hoc* hypotheses to explain specific situations. And finally, such a strategy for the naturalization of landscape often underestimates the role of cultural and sociological elements in addressing the health-landscape connection.

#### Attention Restoration Theory (Psychology)

*The Experience of Nature* (Kaplan and Kaplan, 1989) examines the qualities that characterize restorative environments, that is to say, environments that help restore our attention. Irritability, anxiety, stress, lack of perception, and lack of interest in human beings have all been recognized as a direct consequence of attention fatigue. Kaplan and Kaplan developed ART (Attention Restoration Theory) on the basis that we can better concentrate and restore our directed attention after experiencing nature, since during that experience we are attracted by an involuntary attention or *fascination* (Kaplan and Kaplan, 1989; Kaplan, 1995; Warber et al., 2013, p. 21).

Taking inspiration from Kaplan and Kaplan (1989, p. 178; see also Kaplan, 1995, p. 178), James (1892) distinguish between directed, effortless and restored attention. When our directed attention needs to rest, “it is necessary to find some other basis for maintaining one’s focus. What is needed is an alternative mode of attending that would render the use of directed attention temporarily unnecessary” (Kaplan, 1995, p. 172). The solution lies in what James called “fascination,” and Kaplan and Kaplan describe as “attention that requires no attention at all, such as when something exciting or interesting happens and we look to discover what is going on” (Kaplan and Kaplan, 1989, p. 179; see also p. 184). The restorative experience is based on a series of specific elements which, in addition to fascination, include “being away” (Kaplan, 1995, p. 174) and a concept of “extent” based on connectedness and scope, implying the sense of being in a whole other world (Kaplan, 1995, p. 175; see also Kaplan and Kaplan, 1989, p. 183). According to Kaplan and Kaplan (1989, pp. 173, 180), natural environments fulfill some of these features: not only do they reduce stress by eliminating directed attention fatigue, they can also help to prevent it. This psychological theory proposes a specific framework, focused on restoration of attention and the involuntary role of fascination in human mind. It is one of the most interesting studies explaining the link between health and nature, even though it does not take explicitly into account the cultural aspect of perception. The majority of the research in environmental psychology follows either ART or stress reduction theory, or both of them (Bratman et al., 2012, p. 122). The main difference between the two relates to the fact that the former is more focused on cognition, the latter on evolutionary elements.

#### Emotional and Ontological Connection with Place (Philosophy/Human Geography)

The majority of philosophical theories of landscape follow a phenomenological approach (the main reference being Merleau-Ponty, 1960, 1964) and are based on the idea that humans are embedded in and determined by place with respect to their cognitive/perceptional and emotional/political processes (Casey, 1993, 1997, 2002; Malpas, 2011). However, one of the most important contributions to this debate came from the field of human geography, when Tuan (1974, 1977) defined the concept of place-attachment in terms of *topophilia*, i.e., humans’ affective ties with their environment.

*Topophilia* (from the ancient Greek *topos*: place and *philia*: love, attachment) means that human feelings, values and attitudes toward the world are geographically “embedded,” implying that experiencing places plays a fundamental role in our development. Tuan was one of the first geographers to provide an understanding of *place* as a product of perceptive and cultural elements: place and perceiver are linked by values, ethical commitments, and feelings. He also introduced the idea that anonymous space is changed into articulated geography through the actions and values of people. His distinction between space and place and his genealogy of the concept of place have become very important for many geographers, philosophers, and sociologists, as well as for cultural approaches to place in general. His idea of *topophilia* has also been studied in connection with well-being, meaning that individual preferences for specific places and restorative environments are significantly associated with quality of life (Ogunseitan, 2005; Ruan and Hogben, 2007).

Several other concepts – which can be considered variations on the theme – such as sense of place, place-identity and place-attachment, were developed from the concept of *topophilia*, and analyzed through both experimental research and philosophical works (Lewicka, 2011). Recently, the concept of *topophilia* has reemerged in relation to *biophilia*. The latter notion is an evolutionary term introduced by Wilson (1984) to refer to the genetic/adaptive/evolutionary affiliation between human beings and nature. The concept has been justified in terms of place-attachment: “The *biophilia* hypothesis proposes the existence of an ancestral adaptation, which drives us to appreciate natural environmental conditions and the living world, that evolved because such emotional drive would ultimately be beneficial for survival and reproductive success” (Beery et al., 2015, p. 8842). The attempt at linking *topophilia* and *biophilia* (Sampson, 2012; Beery et al., 2015) relies on an idea of landscape grounded in both cultural and biological-evolutionary concepts, with benefits for human health and implications for landscape management. The role of the concept of *topophilia* and its variations has been widely recognized in the literature (Lewicka, 2011). Its recent developments demonstrate also that there is a need to connect cultural approaches to place-attachment with naturalized ones.

### Evidence of the Health-Landscape Relationship: Toward a New Definition of Health

Over recent decades much evidence – mostly medical – of the health-landscape relationship has been provided. The main theory on which this evidence is based and toward which it leads is known as SDH theory. In this section we analyze the principal medical evidence reported, summarize SDH theory and attempt to provide a new definition of health, based on notions of both agency and landscape. The SDH approach is quite interesting in that it defines a broader concept of health, yet we feel that the other part of the process, i.e., landscape, is missing. The idea of *processual* landscape aims to resolve this problem.

The late 1990s and the early years of the new millennium witnessed mounting evidence in support of the necessary relationship between health (and well-being) and landscape. This evidence prompts us to question the concepts of care, health and place of care, as well as the role of the patient him or herself. In other words, it prompts us to question the relationship between medical *structure* and *agency*.

One of the first piece of evidence attesting to the landscape-health relationship is the idea of *therapeutic landscapes* (Gesler, 1992, 1993; Williams, 2007), a term used in environmental psychology and health geography to denote those restorative places/spaces that provide treatment or healing or that, more generally, restore, improve and maintain health and well-being (Milligan and Bingley, 2007, p. 800). The idea of *therapeutic landscapes* can apply to a wide variety of landscapes: from the unique and specific ones to ordinary scenes. The category encompasses national parks, local urban landscapes, gardens (Milligan et al., 2005), social forestry and woodland (Ward Thompson et al., 2004; Milligan and Bingley, 2007) and even hospitals or asylums located in the countryside. Moreover, the idea of therapeutic landscape implies the improvement of the medical community, specifically in terms of the relationship between health structure and agency (Gesler, 1992).

Recently, the concept of therapeutic landscapes has been broadened to include some other symbolic terms referring to place-attachment. However, despite its different meanings and applications, the notion of therapeutic landscapes allows us to question the idea that physical and mental health problems are merely personal issues which should be addressed solely through individual-based interventions (Wood et al., 2015). It also adds a chapter to the nature/nurture debate and fosters an awareness of the fact that environment and health are necessarily interconnected or, more radically, that nature/nurture is a false dichotomy, since “we hold that environmental and cultural components of health care are inseparable” (Gesler, 1992, p. 737).

Many other studies also present ample medical evidence (both direct and indirect) of the health-landscape relationship. These range from the aforementioned psychological and stress-related studies (see section on Research Focused on Restorative Environments), to ones connecting obesity and frequentation of parks and those relating respiratory/cardio-vascular diseases and green spaces in the prevention of or recovery from specific health conditions (Frumkin, 2003; Keniger et al., 2013; Shanahan et al., 2015). There is also a relevant debate about the dose of or degree of exposure to green space/parks/landscape that we need in everyday life to maintain and preserve our health (Barton and Pretty, 2010; Shanahan et al., 2015, p. 477). The broader climate change discussion includes an attempt to connect biodiversity and medicine: the thesis advanced in this respect is that we need to protect global biodiversity and landscapes because the majority of the medicines we use come from natural resources (Butkus, 2015).

Thus, health practitioners and policymakers not only recognize the impact of place and physical and social environments on health and well-being (Hordyk et al., 2015), they actively stress the role of landscape in the improvement of health and call for nature-based health interventions (Shanahan et al., 2015, p. 482).

All this evidence led to the drafting of the UN document on Commission on Social Determinants of Health [CSDH] (2008) and World Conference on Social Determinants of Health (2011) and to the SDH approach (Solar and Irwin, 2010), which can be considered both further evidence of the relationship between health and landscape and, better still, as the final result of a decades-long struggle. SDH is the study of the full set of conditions under which living takes *place*, and their impact on health. Both the document and the consequent approach link health to a number of elements, including (among others) governance, environment, education, employment, social security, food, housing, water, transport, and energy. Most importantly, the UN document calls for healthy places for healthy people (Commission on Social Determinants of Health [CSDH], 2008, chapter 6), stating that: “Where people live affects their health and chances of leading flourishing lives” (Commission on Social Determinants of Health [CSDH], 2008, p. 60) and “communities and neighborhoods that ensure access to basic goods, that are socially cohesive, that are designed to promote good physical and psychological wellbeing, and that are protective of the natural environment are essential for health equity” (Commission on Social Determinants of Health [CSDH], 2008, p. 60). It is thanks to the UN document on Commission on Social Determinants of Health [CSDH] (2008) and World Conference on Social Determinants of Health (2011) that we can provide a broader definition of health and well-being. Health is thus considered as being socially determined. However, in order to avoid falling into the trap of environmental determinism (Blacksher and Lovasi, 2012), it is necessary to specify the role played by the concept of agency.

The common-sense meaning of health refers to the biological realm and implies the absence of a pathological condition (both physiological and psychological). Nevertheless, already in the preamble to the Constitution of the World Health Organization (1948), health is defined as a state of “complete physical, mental and social well-being and not merely the absence of disease or infirmity.” This definition was questioned and criticized in many ways, for the absoluteness of the term “complete,” as well as for the fact that in this definition people with chronic diseases are considered definitively ill (Huber et al., 2011, p. 1). To date, the concepts of health and disease have been questioned by several philosophical approaches, including naturalism/constructivism; instrumentalism and comparativism (Murphy, 2015). Yet contemporary definitions of health are focused on both the notion of agency (e.g., skills and adaptive capacities), and on social-environmental determinants. Only by combining these two elements is it possible to avoid the opposition between “life chances and life choices” in determining health (Blacksher and Lovasi, 2012, p. 173).

If we take agency into consideration, health should in fact be “based on the resilience or capacity to cope and maintain and restore one’s integrity, equilibrium, and sense of wellbeing” (Huber et al., 2011, p. 2). Health connects to the strategies of the agent/patient/human being, to their autonomy and capacity to adapt and self-manage – physically, mentally and socially (see also Nordenfelt, 2007; Sturmberg, 2013; Sturmberg and Lanham, 2014). From this perspective, a definition which integrates both agency and environment could be: “Health is a state of wellbeing emergent from conducive interactions between individuals’ potentials, life’s demands, and social and environmental determinants” (Bircher and Kuruvilla, 2014, p. 368). In this case, health and the space of the agent are considered as inseparable concepts; accordingly, determinants of health can thus be divided into: (1) individual, (2) social, and (3) environmental. Health, therefore, “occurs when individuals use their biologically given and personally acquired potentials to manage the demands of life in a way that promotes well-being. This process continues throughout life and is embedded within related social and environmental determinants of health” (Bircher and Kuruvilla, 2014, p. 369).

In this scenario, the pitfall of environmental determinism is overcome by specifying and analyzing the culture and perceptional preferences of the agent (Blacksher and Lovasi, 2012, p. 174), their psychological history and, most importantly, their landscape. Although the concept of SDH may risk appearing somewhat vague and overly broad, the notion of landscape, on the other hand (of which we offer both an ecological and cultural definition), is an appropriate and specific tool for describing the relationship between our body, our health, our well-being and the space around us. We can reformulate the *structure-agency* binomial pair into the *landscape-perceiver* one. We will explain the rich concept of landscape in the following section, before offering a definition of processual landscape which will finally be used to comprehend the notion of health and propose a theoretical framework to account for the health-landscape relationship.

## What is Landscape? The Cultural Approach and Beyond

In this section of the paper we provide a comprehensive definition of landscape which connects both the cultural and ecological dimensions. We consider it one of the key factors for recognizing the role of landscape in determining well-being and health.

When we talk about landscape several features are implied: cultural, visual, artistic and, we hold, ecological and naturalist. The following paragraphs aim to explain and connect all these dimensions through the bridge concept of affordance. We also demonstrate that landscape can be distinguished from other similar concepts such as space, place and territory, due to its perceptual implications and, in particular, its aesthetic and therapeutic qualities. *We live embedded in landscape* and, as we add, *we perceive it through our whole body*, and therefore *it affects our well-being*. This is why we need a more comprehensive definition of landscape.

During the last decades of the 20th century, the so-called “spatial turn” (Gould, 1996; Warf and Arias, 2009) in human sciences substantially increased the role of landscape theory in philosophy, sociology, anthropology, human geography, and geophilosophy (Menatti, 2011, 2013, 2014b, 2015a,b). Thanks to pioneering contributions by a large number of scholars (Relph, 1976; Tuan, 1977; Berque, 1995; Roger, 1995; Casey, 1997; Malpas, 2006), the current aesthetic meaning of landscape goes beyond the modern idea of landscape as a view or postcard. Landscape is thus considered a cultural product which refers to every kind of place and space: “Landscape is an important part of the quality of life for people everywhere: in urban areas and in the countryside, in degraded areas as well as in areas of high quality, in areas recognized as being of outstanding beauty as well as everyday areas” (Council of Europe, 2000: Preamble).

Historically, one of the first cultural definitions of landscape stems from the French philosophical tradition (Berque, 1995, 2006; Roger, 1995, 1997; Paquot and Younès, 2009; Bonnaud and Younès, 2014) and differs from a sociological interpretation of the concept. Landscape is not just the idea we have of it (what sociology calls “collective representation”), but rather encompasses also the practical actions performed on the environment in order to adapt the actual landscape to the representations produced by society. In other words, landscape implies at the same time both reality and the appearance of reality (Berque, 1995, p. 16). This means that landscape can be defined as something real, as constituted by actual objects (something out there), but at the same time it can be described as an appearance, insofar as it is a product of our way of seeing, depicting and painting it.

It is important to specify, however, that before being cultural, landscape was (and still is) considered something visual and connected to the figurative arts. From an historical point of view, the European notion of landscape was born within painting, and only as a feature of the Western Modern age. The French research tradition on landscape affirms that some societies of past centuries were not landscape-aware, and the emergence of the concept of landscape is a specific characteristic of Ancient China – almost two thousand years ago – and Modern Western Europe (Berque, 1995, 2008). For example, it is impossible to find any reference to it in ancient societies, such as, for example, Ancient Greek society, especially in philosophical and literary sources.

The etymology of the term seems to confirm its visual origin: the word “landscape” (*landskap*) first appears in European languages in around the 15th century, in the Netherlands. The suffix -*scape* (and then the verb “to shape”) designates something showing, exhibiting or embodying a quality or a state. In the 16 and 17th centuries, “landscape” meant a picture representing natural inland scenery (Pungetti, 1996). Thus the term appears in literature after painters had already discovered it. This idea that landscape is a portion of the Earth that can be viewed from one spot is shared by several authors (Cosgrove, 1984–1997; Jackson, 1984; Cresswell, 2004).

The history of the emergence of landscape demonstrates that it is a visual/pictorial/cultural concept, introduced in a certain period of human history. It is a term that conveys a specific meaning from an artistic point of view, but which also implies a precise idea of space, embedded in a specific culture. If we chart the history and development of the concept of landscape, we see how, over recent decades, it has become synonymous with “place,” involving characteristics such as identity, history and memory^1^.

As Jackson (1984, pp. 3, 5, 8, 156) wrote in *Discovering the vernacular landscape*, originally, the term did not even mean a view, but rather a picture of it, “an artist interpretation.” Yet the definition of landscape has evolved to become a complex entity that is always *synthetic*: it is not a natural feature, but rather a synthetic space, a human-made system of spaces superimposed on the face of the land. And because that system is made by the community and for the community, landscape is not fixed once and for all; it is always subject to sudden or unpredictable changes.

Indeed, landscape (as a way of looking at the land) assumes different meanings. Social and cultural elements are considered integral to it, constituting a “discourse” through which social groups frame themselves and their relationship with both the territory and other social and political groups. “The landscape idea represents a way of seeing – a way in which some Europeans have represented to themselves and to others the world about them and their relationship with it, and through which they have commented on social relations. Landscape is a way of seeing that has its own history, but a history that can be understood only as a part of a wider history of economy and society” (Cosgrove, 1984–1997, p. 14).

The history of the concept of landscape moves forward from the idea of vision (Shapiro, 2003), or artistic representation, to landscape as a cultural product.

To date, the main definition of landscape remains the cultural one, which strikes a balance between aesthetics and memory, perception and place-attachment, history and no-places. The majority of analyses are still indebted to the artistic/visual definition. Nevertheless, the most important aspect (or perhaps the aim) of the cultural definition of landscape is that it moves beyond the *postcard* effect: the perception of landscape cannot be considered something natural or naïve; rather, it depends on a specific cultural disposition and education.

Recently, the Council of Europe (2000) has attempted to provide an even more comprehensive definition of landscape, incorporating steps toward defining the political and ethical aspects of this concept. The ELC acknowledges that landscape is not simply a view, but also a place with its own cultural and social dimension (see also Howard, 2004, p. 425). Most importantly, the Convention overcomes the previous aesthetic distinction between beautiful and ugly places, between outstanding landscapes and those simply not worth considering. Its main innovation is the idea that *everything is landscape*: ugly areas as well as beautiful ones, urban surroundings as well as rural ones. According to the ELC, all landscapes contribute to the formation of local culture, for good and for bad, and all have an impact on human well-being and the consolidation of European identity. Landscape is thus conceived as: “an area, as perceived by people, whose character is the result of the action and interaction of natural and/or human factors” (ELC: Chapter 1, General Provisions, Article 1. Definitions). Its study and management requires both cultural and ecological approaches. A landscape has its own specific identity, which has to be safeguarded and protected, but at the same time it requires cross-border cooperation at the local and regional levels to prepare and implement joint landscape programs (Council of Europe, 2000).

Although the ELC also includes elements that allow us to consider landscape as a common good and even a human right, this topic falls beyond the scope of this paper (see Egoz et al., 2013; Menatti, 2015c). What we have attempted to provide in this section is simply a brief history of the concept of landscape from a visual, perceptional, and cultural point of view. Nevertheless, in order to avoid the risk of separation and to bridge the gap between social and natural sciences in their approach to this concept, we believe it is necessary to take a step forward in the naturalization of landscape and make an original contribution. That is what we will attempt to do in the next section.

### A Step Toward Biology and Ecology: Naturalization of Landscape

The next step in providing a broader account of landscape is its naturalization^2^ in connection with ecology. This link with the natural and behavioral sciences (biology, medicine, psychology, and ecology) is still under-theorized. As of 11/11/2015, a search in the Frontiers website with the keywords “landscape AND psychology” provided 39 results, but only one of them was relevant for the purposes of establishing a connection between landscape and psychology in naturalistic terms (Lin et al., 2014). There remains, in fact, a sharp separation between those who study landscape as a physical environment and those who study it as a social and cultural product. We, on the other hand, argue that the study of landscape both as a cultural product and an ecological process should be considered a priority, both for its definition and for its management.

Two main questions are involved in this debate: (1) How do we perceive landscape? (2) What actually is landscape? These questions are intertwined and their answers have repercussions for our health and should be analyzed in accordance with a cultural/naturalist approach.

Some earlier arguments put forward as part of this debate may prove clarifying in this respect. Some authors have successfully tried to connect aesthetics and ecology (Bourassa, 1990; Naveh, 1991, 2005; Nassauer, 1995, 2011, 2012; Carlson, 2000). Specifically, these authors are architects and ecologists who believe in a concept of landscape which broadens the range of and possibilities for its management. Their thesis is based on the acknowledgment that landscape is modeled from the social interactions which produce new and always adaptive aesthetic, cultural and ecological definitions. For instance, Daniel (2001) argues that visual landscape quality assessment (in the 21st century) is a product of the relationship between different perspectives: expert and designer parameters, sensory and perceptual parameters and cognitive constructs (Daniel, 2001, p. 268). These three kinds of parameters reflect (respectively) three definitions of landscape: landscape as a view, as a culture-rich environment and as a portion of territory considered the prerogative of ecologists, architects, and other specialists. When we have to operate on/manage landscape, all these aspects should be considered and merged together. Yet it seems that, until now, ecological values and socio-cultural paradigms have been opposed. Contrary to this attitude, the goal of a new landscape management model would be to successfully merge these two approaches, since it could “better serve environmental managers and insure a more effective representation of visual aesthetic quality in management decision and policies” (Daniel, 2001, p. 278). “How do we perceive landscape?” becomes the fundamental question. The answer is always complex, but our ways of perceiving landscape surely influence how we produce, create and dwell in it.

Similarly, Nassauer (1995, p. 230), taking inspiration from Jackson’s (1984) definition of landscape as a “synthesis,” proposes closer collaboration between aesthetics and ecology in the field of architecture and design: “The focus has been on landscape structure, not on human behavior. A more functional perspective quickly demonstrates that humans not only construct and manage landscapes, they also look at them, and they made decisions based upon what they see, and know and feel” (Nassauer, 1995, p. 230; see also Gobster et al., 2007). The perception of landscape thus assumes a pivotal role.

This brief excursus shows the effort which has been made by ecologists and architects to consider the cultural and perceptional side of landscape, while for their part, those working in the humanities have attempted to ground landscape theory in biology and evolution (as outlined in the previous sections). A pivotal role in this debate has been played by aesthetic preferences, i.e., how we choose a beautiful landscape, why we prefer one landscape to another, and for our purpose, how a landscape could be better or worse for our health and well-being. Naturalization through evolution is one possible strategy. However, it risks relying on *ad hoc* answers; for instance, drawing on Dewey’s (1934) ideas, Appleton (1975) argues that the origin of aesthetic values is to be found in natural evolution as understood by the life sciences. Human evolution from hunters has provided us with a preference for prospects and refuges which allow us to “see without being seen.” The objects of our landscape, whether seen directly or experienced through the medium of the painting, are symbols of values developed throughout centuries of biological evolution. Appleton analyses major forms of art (including poetry and painting) in terms of being refuge-dominant or prospect-dominant landscapes. His theory does not exclude the idea of culture in the development of aesthetic values, but connects cultural landscape theory with ecological and ethological theses.

Appleton’s theory brings us to another core point of the debate: the naturalization of art. In art theory (as well as in the debate about our aesthetic preferences) there is major opposition between culture-based theories of perception (see Di Summa-Knoop, 2014, p. 193) and naturalist or neuroscientific perspectives (Seeley, 2006; Brown et al., 2011; Prinz, 2011; Chudoba and Wilkoszewska, 2015). The naturalization of art implies that in perceiving and producing art we mostly follow evolutionary and adaptive bases. Evolutionary psychology and art theory based on sexual selection and genetic inheritance share a transversal and inclusive concept of art, and they argue that art has been present in all cultures. There is and always has been art in all societies, because it allowed human beings to survive and reproduce (Dutton, 2009, p. 64). With respect to the perception of landscape, Dutton specifies that “people in very different cultures around the world gravitate toward the same general type of representation: a landscape with trees and open areas, water, figures, and animals. More remarkable still was the fact that people across the globe preferred landscapes of a fairly uniform type” (Dutton, 2009, p. 14). By referring to Komar and Melamid’s studies and artwork (Dutton, 2009, p. 15; Herrington, 2009, p. 23), which showed a cross-cultural preference for the color blue in landscapes, as well as for water and other features recently depicted in the famous painting *America’s most wanted^3^* (Wypijewski, 1999), Dutton infers that landscape preferences are innate and are the expression of natural atavism. He states, in fact, that “This fundamental attraction to certain types of landscapes is not socially constructed but present in human nature as an inheritance from the Pleistocene, million years during which modern human beings evolved. The industry has not conspired to influence taste but rather caters preexisting, precalendrical human preferences” (Dutton, 2009, p. 18). According to this theory, the origin of landscape preference relies on a combination of the aforementioned studies by Appleton (1975) and evolutionary psychology as developed by Ulrich (1983) and Kaplan (1987). Moreover, Dutton refers to the famous savannah hypothesis on landscape tastes (Orians and Heerwagen, 1992; Falk and Balling, 2010), which is based on the idea that, like the hominids in the African savannah, human beings continue to prefer open, mildly flat landscapes (savannah-like settings), with water directly in view and a clear way both to avoid predators and to keep an eye on them. Dutton emphasizes that this kind of explanation of landscape tastes implies that the cultural background plays a minimal or null role.

Yet the naturalization of art does not necessarily always deny the role of culture and cultural background in aesthetic appraisal. Naturalism generally implies an attempt to ground its conclusions in empirical findings. Moreover, although many neuroscientists and philosophers of art follow only psycho-evolutionary theory, others argue that emotions, which play a pivotal role in aesthetic appraisal, may be different in different cultural settings (Prinz, 2011, pp. 71, 75).

Likewise, we exclude neither culture nor naturalization from the determination of perception of landscape, and consequently from its definition. On the contrary, we believe that a broader and more comprehensive theory of landscape will prove a key factor in explaining why there is such a close and far-reaching relationship between health and landscape. If it is agreed that cultural background may determine the psychological and emotional well-being of the agent, then it is the naturalization of landscape which is able to explain its connection with medically understood health.

In the end, to naturalize landscape without denying its cultural dimension, we need to focus on how we perceive it. Perception entails both our role as “agent” (thus our cultural determination in perceiving, and our mind and physical body moving in, the landscape) and the idea of “structure,” i.e., our relationship with the ecological environment in which we live. In order to understand what landscape is and how it affects our health, we have to address biological and ecological theories of perception of the environment.

**FIGURE 1:**
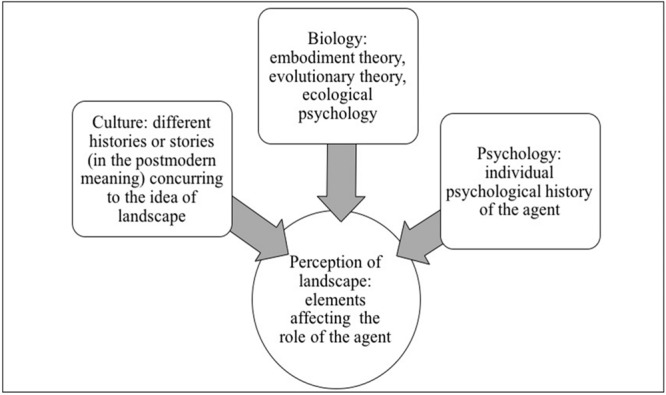
**Perception of landscape**.

**FIGURE 2:**
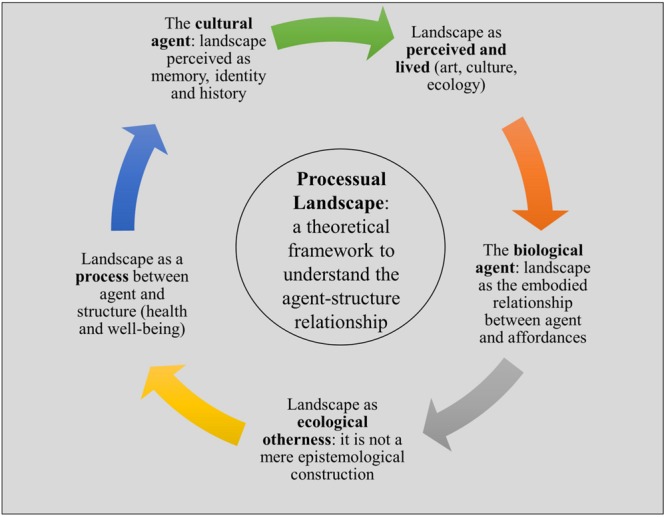
**Processual landscape**.

The perception of landscape is a complex scheme insofar as we participate in the construction of our environment. We propose to firstly focus on the role of the agent and to consider landscape as the product of a group of histories. To visualize these histories, we have drawn up a simple diagram (**Figure 1**) which will be further developed in our concept of a processual landscape (**Figure 2**). As **Figure 1** shows, landscape can be analyzed as a product of cultural history (or “stories”), subjective history and biological history. We take inspiration from Berque (1995), according to whom different scales participate in the construction of landscape through human perception. This author also adds that landscape is a *médiance* (a medium) between the material (objective) and the ideal (subjective; Berque, 2000).

The biological dimension – both the agent’s and the landscape’s, both the perceiver’s and the structural- allows us to introduce and clarify the relationship between health and landscape. The biological dimension implies different approaches: the evolutionary, the ecological and the embodiment theory of knowledge and perception. The embodiment theory of perception will be analyzed using Gibson’s theory^4^. While the analysis of the cultural origin of landscape and its visual dimension is now clear (see Introduction and Main Theoretical Issues and Approaches), we have yet to demonstrate how the concept of landscape could be naturalized through a theory of perception. Even though naturalization has already been attempted by both evolution-based and neurophysiology-based approaches, the theory we favor when analyzing landscape perception is J. Gibson’s ecological psychology (Gibson, 1979).

Our choice is motivated by the following reasons:

• To date, Gibson’s theory has proven effective in describing perception according to a framework which is not merely stimulus response based.
• It connects perception with the agent’s “here and now.” It addresses the actions and the actual event of perceiving, rather than focusing on the evolutionary history of the agent, which might incur the risk of epiphenomenalism (Christensen and Bickhard, 2002).
• It does not reduce perception to a merely neurophysiological process.
• It is one of the few psychological theories which establish a connection between ecology and psychology.
• Even though it is mostly focused on visual perception, the motor perception interpretation overcomes this limitation and introduces the idea of embodied perception of space.
• Gibson’s theory solves the problem of environmental determinism thanks to the role played by the perceiver in their relationship with the *affordances* (as we will explain in the next section).

## Ecology of Perception: A New Role for the Perceiver

The expression “ecology of perception” was introduced by Gibson (1966, 1979, p. 41) as an approach to visual perception in which the latter is not reduced to the S/R dynamic between stimulus and responses, but rather consists of the relationship between the affordances of the environment and the perceiver, in an interconnection in which the philosophical dichotomy between subject and object becomes obsolete. By questioning the classical idea of perception, we argue, Gibson questions the idea of landscape as well.

Although the application of Gibson’s theory to landscape studies is not new (Heft, 2010), it has mainly been done in the field of design and landscape architecture, rather than in applied psychology or healthcare. But in both cases the application implies nothing less than a change of paradigm. According to Heft (2010), research into perception and aesthetics in environmental psychology has long been dominated by a concept of vision that can be traced at least as far back as the Renaissance. In this old paradigm, vision is considered the primary element in defining landscape and is conceptualized as an image-capturing process (Heft, 2010, p. 10). Gibson’s approach to perception is radically different from both this older paradigm and the traditional cognitive science that accompanied it (Escribano, 2012, p. 48–50). Classical ideas of perception (those which are based on the stimulus as a retinal input) conceive the perceiver as stationary, “positioned like a camera at a fixed observation point. From this perspective, the perceiver is ‘at a remove’ from the perceived, adopting the stance of a detached spectator rather than that of an engaged agent” (Heft, 2010, p. 11). Gibson, the other hand, introduces a new theory of perception by asking what role perceiving plays in the everyday functioning of an organism. Perceiving becomes a part of the ongoing processes that make up the life of an organism, and is necessary to its survival. Moreover, in this view, perceiving and acting are part of the same process and are fundamental to sustaining life in nearly all complex organisms: “Observation on living things reveals that organisms are continually active, turning their head, moving around while looking, as well as felling, sniffing, tasting […] Perceiving, and acting are intertwined. Indeed, separate terms ‘perception’ and ‘action’ can be somewhat misleading” (Heft, 2010, p. 14–15).

Gibson’s theory shows that there is not just one vision (only one perceptive resultant), there is not only the aperture vision, concerning the basic configuration of the object, as if we were looking at something through a hole in a fence. Real vision also concerns the free movement of the head (ambient vision) and the free movement of the body (ambulatory vision). Looking around and getting around do not fit into the standard idea of what visual perception is and, according to Gibson (and to the experiments he conducted on the optic ambient array rather than on fixed vision, or “snapshot vision”), visual awareness is actually panoramic and persists during long acts of locomotion.

Furthermore, the perceiver finds himself emplaced with a body in an ecosystem. This is the fundamental step through which it is possible to exit the “picture world” and enter the world of ecosystems, of organisms and, in this specific context, we add, the world as constituted by different landscapes. As Ingold puts it, we perceive “not from a fixed point, but along what Gibson calls a ‘path of observation,’ a continuous itinerary of movement […] if perception is thus a function of movement then what we perceive must, at least in part, depend on how we move. Locomotion, not cognition, must be the starting point for the study of perceptual activity” (Ingold, 2011, p. 46).

Ingold also points out an important connection: the idea of a perceiver embedded in the world was developed from a philosophical point of view by (Merleau-Ponty, 1945, 1960, 1964), and then pursued by Gibson’s theory from a psychological perspective. There are differences between the two frameworks, but they share a non-objectivist perspective of vision. Ingold compares the way in which the French philosopher describes the perception of the painter with the way in which Gibson analyses our perception: “The painter’s relation to the world, Merleau-Ponty writes, is not a simple ‘physical-optical’ one. That is, he does not gaze upon a world that is finite and complete, and proceeds to fashion a representation of it. Rather, the relation is one of ‘continued birth’ – these are Merleau-Ponty’s very words – as though at every moment the painter opened his eyes to the world for the first time […] His vision is not of things in a world, but of things becoming things, and of the world becoming a world” (Ingold, 2011, p. 27).

In addressing the problem of perception in opposition to classical cognitivism and empiricism, Gibson developed a non-representational idea of cognition that nevertheless occurs directly through what he calls the “direct pick up of information” (Gibson, 1979, p. 238), and which is embodied and integrated in the environment. Gibson strongly argues against indirect perception^5^ and claims that an organism directly perceives meaning from the environment and that this happens at the level of medium, surfaces, substances, and events that are relevant to its life.

### *Affordances* as the Possibilities Offered to Living Organisms

In order to specify the moment of perception and exemplify the relationship between perceiver and medium, Gibson introduces the concept of *affordance*: in the flow of perception the human perceiver directly picks up affordances. In elaborating this concept, he is in debt to Gestalt psychology, specifically the figure/ground relationship: elements are perceived as either figures (distinct elements of focus) or ground (the background or landscape on which the figures rest). It is in this context that a series of illusions are usually presented as the basis of Gestalt theory. They demonstrate how visual perception is determined by a non-mediated reconstruction of the context of the stimuli. For Gestalt theory, the meaning or the value of a thing seems to be perceived just as immediately as its color. Actually, Gibson follows one of the creators of Gestalt psychology, Koffka (1936), when the latter says that “fruit says eat me; water says drink me; thunder says fear me [...] the things in our environment tell us what to do with them” (Koffka, 1936, pp. 7, 353).

What is an *affordance*, then? The first definition given by Gibson is: “What the environment offers the animal, what it provides or furnishes, either for good or ill [...] something that refers to both environment and the animal in a way that no existing term does. It implies the complementarity of the animal and the environment” (Gibson, 1979, p. 127). Using this new term, Gibson describes what environment *affords* to animals: terrain, water, fire, objects, tools, and other animals. The crucial point is to understand how the environment affords (that is, gives the possibility of) perception and action. The composition and the layout of surfaces constitute what they afford, and affordance emerges only when different characteristics of individuals, such as their physical dimensions and abilities, social needs and personal intensions, are *matched* with the features of the environment.

According to a simplified interpretation of Gibson’s theory, affordance is usually considered as a property of the natural environment, offered to organisms in action. There is no shortage of interpretations of Gibson’s work (e.g., Heft, 1989, 2001, 2010; Turvey, 1992; Reed, 1996; Michaels, 2000, 2003; Stoffregen, 2000), some of which are related to design and ergonomics (Norman, 1988; Xenakis and Arnellos, 2012). Affordances are mostly considered properties or resources of the environment that have significance for the animal’s behavior. However, the definition of affordance is more complex, as it implies the concept of relation: “Affordances are controversial perceptual objects, composed of both subjective and objective components that are formed from the direct perception of environmental invariants that are of relevance to the individual. Instead of perceiving an object environment, and then determining some future action through hypothesis testing over a symbolic world model, we directly perceive what the environment affords given our own physical embodiment. It is then the fulfillment of these affordances that enables us to sense and act in a continuous environment” (Worgan and Moore, 2010, p. 331).

This relational character of affordances implies that they are not properties of either the environment or the animal. Affordances, Chemero (2003, p. 185) argues, are “relations between particular aspects of animals and particular aspects of situations.” So, first of all, affordance is neither a property, an a-priori concept, nor a universal measure. It is something unique for every animal and it belongs to (and emerges within) the relationship between the environment and the perceiver. Different layouts afford different behaviors for different animals, with mechanically different encounters. The different ingredients of the environment have different affordances for nutrition and manufacture; different objects have different affordances for manipulation. Also, human and non-human beings reciprocally afford each other a complex set of interactions. Finally, affordance is related to movement: through the affordances of the environment, the body in movement perceives the main invariants of said environment. This is important, since it means that Gibson’s theory is related to ecological perception on the basis of agency and the actions performed by an agent or animal.

According to Mossio and Taraborelli (2008), Gibson shares with other authors a sensorimotor approach to perception, meaning that the coupling between motion and the senses is the key to understanding perceptual phenomena: there is no fixed perception such as the postcard view. Moreover, the role of action is basically adaptive, meaning that “the animal perceives affordances in the environment that allows them to attain specific goals” (Mossio and Taraborelli, 2008, p. 1335).

Gibson (1979, p. 130) also points out that it is impossible to separate the cultural environment from the natural one, as if there were a world of mental products distinct from the world of material ones: “There is only one world, however, diverse, and all animals live in it, although we human animals have altered it to suit ourselves.” Our hypothesis is that this concept of affordance can be used to create a bridge between cultural landscape theory and ecological thought, by introducing a naturalized approach to landscape which takes into account the cultural dimension of our environment. We could even associate landscape itself with the affordances if provides (cf. Berque, 1995), i.e., the perceptional characteristics that enactively emerge during the interaction between the environment and the perceiver (for a discussion of interaction affordances, see Worgan and Moore, 2010).

In the recent debate about affordance and perception, Rietveld and Kiverstein (2014) reject the idea that affordances are features of the environment or mechanical elements with ergonomic possibilities. Instead, by drawing on Chemero’s relational approach and other important contributions (Costall, 1999; Heft, 2001), they relate affordance to the specific skills of a human or non-human animal. Rietveld and Kiverstein (2014, p. 326) propose a new framework based on the idea that affordance always involves the exercise of an *ability* in a specific context: “We argue that the affordances the environment offers are dependent on the abilities available in a particular ecological niche.”

The main point of this approach is that every affordance is related to specific skills and, in the case of human beings, to sociocultural practices. For this reason, Rietveld and Kiverstein introduce Wittgenstein’s concept of “forms of life” (see Wittgenstein, 1993), by relating the idea of affordance to that of normativity. Actually, when offering a definition of the term “niche,” Gibson himself introduced the idea that each species of animal has its own distinctive way of life: “The niche refers more to how an animal lives than to where it lives” (Gibson, 1979, p. 128; see also Chemero, 2003, p. 192; and Chemero, 2009). Based on this quote, the authors imply that the environment may offer “many ways of life” to different species of animal (Rietveld and Kiverstein, 2014, p. 328).

Seen this way, affordances are located in the context of a form of life. According to Rietveld and Kiverstein, affordances are “possibilities for action the environment offers to a form of life, and an ecological niche is a network of interrelated affordances available in a particular form of life on the basis of the abilities manifested in its practices – its stable ways of doing things. An individual affordance is an aspect of such a niche” (Rietveld and Kiverstein, 2014, p. 330). Human beings embedded in a landscape of affordances develop specific skills though their capacity to distinguish what is correct, optimal, adequate or inadequate. The authors call this type of normativity “situated normativity,” thus reformulating Chemero’s relational character of affordance: “Affordances are relations between aspects of a material environment and abilities available in a form of life” (Rietveld and Kiverstein, 2014, pp. 332, 335).

Concerning the cultural and biological aspects of affordances, Rietveld and Kiverstein consider that the most important thing is the practice in which an ability is embedded. Affordances have an existence that is relative to a form of life. So do landscapes, we might add; both are dependent on a specific form of life (niche) and on the material environment which allows our sociocultural practices: “First the existence of an affordance does not depend on the active use by any particular member of a form of life. Affordances as relational properties depend for their existence both on aspects of the material environment and on the abilities available in a form of life. Second, our practices themselves are dependent on the opportunities for action offered by the material environment, in particular on the causal properties of things we put to use in the services of our projects and concerns” (Rietveld and Kiverstein, 2014, p. 340).

## Processual Landscape: A Framework Connecting Health and Landscape

We see the theory of affordance as fundamental to our proposal of a landscape incorporating both naturalistic and ecological features in our perception of the space around us. The theory developed and described so far in this paper implies important philosophical consequences. The factors described up to here constitute the main pillars of a theoretical framework which we call “processual landscape.” To briefly summarize the main points which allow us to build up a comprehensive idea of landscape as a process, it is necessary to consider (see **Figure 2**):

• The perceiver of landscape as a cultural agent. Cultural theory considers landscape to be a cultural product, in which the perceiver plays an important role in determining landscape through their collective history, personal stories and, in short, cultural background. The relationship between landscape and the cultural perceiver is based on the recognition of cultural invariants of landscape, which are considered important in order to safeguard places and space. The cultural approach analyses the emergence and history of the concept of landscape from its pictorial and visual beginnings (in 15th century Europe) right up to the evolution of a more contemporary and comprehensive idea of a perceived landscape existing in close relation with the life of its inhabitants. Contemporary literature on landscape moves beyond the mere visual definition of landscape to provide a more comprehensive view in line with the Council of Europe (2000). Specifically, we agree with the main contribution of the Convention: landscape is not just outstanding places, but rather includes everyday environments also.
• The perceiver of landscape as a biological agent. The perceiver is also a body that moves in the landscape and is in motion during perception (this is one of the main assumptions of Gibson’s ecological perception). The idea of a perceiver as a whole body originally stems from phenomenology, in particular Merleau-Ponty. Here, however, we focus on the role of the physical agent through ecological perception, which introduces a more complex approach both in the theory of perception and the definition of landscape. In this sense, landscape becomes the embodied relationship between the agent and the possibilities offered by the environment to the living organism. Thanks to ecological psychology and to the consequent theory of affordance, it is possible to naturalize landscape and to introduce the contributions from the scientific domain into a literature that has remained for decades merely cultural. Thus, in this more comprehensive perspective, landscape is not just the cultural product of our society, but rather also the result of our ongoing encounter with ecology and the physical realm, which has traditionally been studied exclusively from the point of view of scientific theories.
• Perception of landscape, according to this new theory, is neither an exchange of information nor a simple stimulus-response relationship. Rather, it is a continuous process of construction, emerging from the mutual determination of the agent (cultural and physical) and the structure (the environment, the landscape and its manifestation as place and space). Perception can be thus understood as a relationship between the perceiver and the affordances, which are the possibilities offered by the environment to the organism, rather than as a mediated elaboration of stimuli. Affordance (Gibson, 1979) allows perception, but also motion, life and survival: it is therefore adaptive.
• Environment, culture and perception cannot be separated: the framework presented here yields a relational approach which moves beyond the classical distinction between organism and nature. According to this view, *affordances* are relational properties: they are the basic invariant in the construction of the built environment and built landscapes. Some authors use the term ‘world of life’ (from Wittgenstein), others *Umwelt* (from Von Uexküll; see also Kull, 2001; Kull and Hoffmeyer, 2005); we have chosen to refer to them as processual landscape in order to underline their dynamic dimension and the two-way nature of the relationship linking the agent and the environment.
• Landscape can be described as a cultural product, but also as an ecological otherness. It is not a mere epistemological construction by human beings. For this reason, the relationship between ecology and aesthetics, humanities and natural science, is crucial if we are to describe landscape as a *process* of codetermination between the agent (the perceiver) and the structure (the environment).

The notion of process is not new in the sociological and philosophical analysis of place and landscape (Lefebvre, 1974; De Certeau, 1984; Massey, 2005), yet the novelty and specificity of the framework we propose here lies in the fact that it integrates most of those elements concurring in the creation of landscape. Our view accounts for the creation of landscape as an interaction between human beings and nature (and its manifestation as place, space, and landscape) that does not give rise to an opposition between the two extremes or a preeminence of one over the other. On the contrary, human beings are characterized as being embedded in the place, in nature and in landscape. In our proposal, therefore, place is seen as a set of *processes* or relationships, thus overcoming both the idea of a mere aesthetic or visual landscape and the idea of a cultural/social landscape that can be simply determined or fully constructed by people, as well as avoiding the pitfall of environmental determinism.

Human beings and landscape are therefore considered as involved in a mutual and dynamic relationship. By basing our proposal on the theory of *affordances* we can state that perceiving the environment (and consequently living in the landscape) is not merely a question of perceiving a value-free physical object to which a meaning is somehow added arbitrarily. Rather, it is a *process* of perceiving/creating a value-rich ecological object. There is no passive place out there: landscape is not fixed and pre-given, but rather a dynamic ecological system, in which both cultural and ecological elements play a constitutive role.

Landscape, we conclude, can be defined as *processual*: i.e., it is both relational and dynamic. Its reality depends on the processes of which it is made up, in the same way as the affordances offered by the environment do not exist without interaction, and yet do not commit us to a purely constructivist approach. Rather, landscapes are the product of the dialectic between culture and the affordances of a place. This relationship is not captured by a realist framework; nor can it be conceived as a constructivist determination. Rather, it is a process in continual evolution, occurring in the interaction between the environment (with the complexity of its affordances and invariants) and the perceiver: a body in motion using its physical and cultural agency (thought and language *are* also motions), in order to establish a relationship with and a boundary for the environment. A processual landscape is what continuously results from this kind of ongoing dynamics, and it generates what we call places, spaces and landscapes.

The integrative notion of processual landscape allows us to take into consideration the connection with health and our well-being on the basis of the simple, yet often under-theorized, idea that we are perceiving organisms (cultural and biological agents) in the environment. As showed in the diagram in **Figure 2**), human agents are an active part of the process of *co-creating* landscape, both from a cultural and naturalist point of view. Their life, body and perception (grounded in the theory of affordance) cannot be detached from landscape. Their mental and physical health depends and relies on it.

The idea that emerges from this processual concept of landscape, supported by Gibson’s ecological psychology, constitutes the framework which allows us to connect health and landscape. The place in which we live is a process interrelating our body/mind and the nature of the environment, in whose creation we are an active agent. Consequently, we cannot discuss our health and well-being without mentioning, considering, and implementing our landscape. In section on Research Focused on Restorative Environments we state that health and the space of the agent are considered inseparable concepts; accordingly, the determinants of health can be divided into three categories: (1) individual, (2) social, (3) environmental. We provided a brief summary of the contemporary definitions of health as a connecting agency and a set of social determinants. In the analysis of social determinants, we consider landscape as pivotal. Unfortunately, current accounts of landscape tend to underestimate the agential dimension, and it is this shortcoming that our framework aims to redress. We propose a way to address the relationship between health and well-being not just through the medical, sociological and psychological evidence produced by decades of studies, but rather through a common factor in the debate about health and therefore about landscape: agency. The agential dimension of the processual landscape provides the missing link with health and well-being.

The concept of agency, firstly developed by Anscombe (1957) and Davidson (1963), currently presents various approaches and different applications in philosophy, psychology, biology, and cognitive sciences (Schlosser, 2015). But the relation between agency and landscape is an unexplored philosophical topic outside Gibson’s theory and the related literature: “The perspective of ecological psychology begins with the recognition of human agency in continuous, reciprocal interaction with the everyday world” (Chawla and Heft, 2002, p. 214; see also Gibson, 1966, 1979; Heft and Chawla, 2006). It is thanks to Gibson, and also to the philosophical phenomenology of Merleau-Ponty, that in this paper the idea of agency can play a pivotal role for the definition of both landscape and health. All human beings involved in the perception of landscape and in its mutual creation can be considered as agents; thus perceiving does not imply a landscape as a collection of given data, but rather it involves an actual experience and interaction (Hamlyn, 1978, p. 545). The importance of the concept of processual landscape proposed in this paper relies, in fact, on a theoretical framework which has overcome the mental/aesthetical/imaginative production of landscape and has introduced the movement of a body acting in the space and which, while it perceives, creates its own milieu. Specifically, within the process concurring to the creation/perception of landscape, agency means physical perception (we perceive through our body and its movements in space) but it implies at the same time the social/cultural creation of place. On the other side, the most recent theories of health affirm that it is agency-based. As we have showed in section “Evidence of the Health-Landscape Relationship: Toward a New Definition of Health,” contemporary definitions of health are focused on both the notion of agency (e.g., skills and adaptive capacities), and on social-environmental determinants (Blacksher and Lovasi, 2012). For this reason, we quoted definitions of health such as (1) “based on the resilience or capacity to cope and maintain and restore one’s integrity, equilibrium, and sense of wellbeing” (Huber et al., 2011, p. 2) or (2) “health is a state of wellbeing emergent from conducive interactions between individuals’ potentials, life’s demands, and social and environmental determinants” (Bircher and Kuruvilla, 2014, p. 368), in order to demonstrate how health connects to the strategies of the agent/patient/human being, to their autonomy and capacity to adapt and self-manage – physically, mentally, and socially. The very idea of our paper consists thus in introducing the term agency in the theory of landscape perception, by naturalizing landscape, even though we do not eliminate the cultural approach to space and place, but rather we broaden and complete it. Agency appears to be the bridging concept between landscape and health: an ecological approach to landscape implies agency; on the other side, a systemic vision of health involves agency.

The aim of this paper is not to produce an exhaustive theory of perception and agency, but to show we become agents when we perceive a landscape. This act is at the same time the result of our cultural background and our direct (and mutual) apprehension of affordances of a place. Every movement thorough which the perception of landscape occurs is agential-based, but at the same time it requires a specific cultural/psychological/emotional background. This aspect, even though it might be controversial in the realism-constructivism debate within Gibson’s theory (Heft and Chawla, 2006), yet it is a theoretically central idea that emerges from our account of processual landscape, that results from the integration of two wide traditions on landscape studies: the cultural and the ecological one^6^.

## Conclusion: A New Theoretical Foundation for Health and Landscape

To sum up, we offer a new definition of landscape as a “processual landscape” capable of explaining why we exist in such a close relationship with this concept. The relationship with landscape based on the concept of culture and affordance (ecological psychology) is the theoretical tool which allows us to understand the bond between health and the environment.

After an overview of the literature on the health-landscape relationship (from the psychological, medical and biological points of view) we propose some fundamental theoretical steps:

• We analyze the cultural concept of landscape, showing that landscape was conceived firstly as a view, then as a picture and finally as a cultural product carrying values such as history, identity and memory.
• We demonstrate that landscape, contrary to the majority of the literature on the theme, cannot be distinguished from the biological and ecological environment.
• We reformulate the notion of landscape by naturalizing it through the concept of affordance and ecological psychology. Here “affordance” means the possibilities that the environment offers to an adaptive organism to enable it to survive and move around. This means that perception is not merely visual, but rather involves our cultural and biological background also. At the same time, while perceiving, the organism builds its own landscape.
• We have chosen to follow Gibson because we believe that a theory of affordance – and its contemporary interpretations – solves the realism/constructivism opposition that emerges when the concept of perception is analyzed. We demonstrate that we perceive because we are organisms moving in a specific landscape that we ourselves build (see also the idea of *Umwelt*). For this reason, our perception and thus our life are connected with landscape. We live in it because we are in a causal relationship with it.

The theoretical framework proposed in this research article implies an agent – an active role in perception – and an environment, which we call “landscape.” In this process human beings are both dependent and co-creators.

The concept of *processual landscape* is therefore the theoretical demonstration of the health-landscape relationship. We propose a framework potentially capable of explaining the reason for our link with landscape. Thanks to medical/psychological/sociological studies, there is nowadays a large body of evidence demonstrating the health-landscape relationship. In order to complete this picture, we need a theoretical framework explaining how the perception of landscape works. Perception is the key to explaining the casual relationship which exists between us and our landscape.

We perceive landscape as we build it, and at the same time we are in a co-determinant relationship with it.

This is the preliminary result of our research, which may be completed by studying the practical consequences of our framework. Insofar as health is greatly affected by landscape, this construction represents something more than just part of our heritage or a place to be preserved for the aesthetic pleasure it provides. Rather, we can begin to talk about the right to landscape (Egoz et al., 2013; Menatti, 2014a, 2015c) as something intrinsically linked to the well-being of present and future generations.

## Author Contributions

LM gathered the main body of data, provided the basic framework of research, draw all the diagrams, wrote the first draft of all sections except Evidence of the Health-Landscape Relationship: Toward a New Definition of Health, and rewrote a full version. ACdR provided additional data, wrote the first draft of section “Evidence of the Health-Landscape Relationship: Toward a New Definition of Health” and rewrote a full version. Both authors discussed the general outline of the article and contributed with comments and revisions.

## Conflict of Interest Statement

The authors declare that the research was conducted in the absence of any commercial or financial relationships that could be construed as a potential conflict of interest.

## Footnotes

1 See, for instance, the difference between place and no-place in Augé (1992).

2 Naturalization concerns the relationship between science and philosophy, and is based on the idea that philosophy needs to follow a scientific method and rely on the resources and results of scientific investigation. The debate arouses in epistemology (Quine, 1969), and has been developed for instance by recurring to neuroscience in philosophy of mind (see Godfrey-Smith, 2003). The naturalization of perception (of landscape) can involve evolutionary theory, theory of mind, evolutionary psychology and, specifically for our purpose, ecological psychology.

3 http://awp.diaart.org/km/

4 Embodiment theory implies a wide range of frameworks. Even though we focus here on landscape perception, we should not forget that embodiment theory was first introduced by Varela et al. (1991) among others, and has recently been developed by Di Paolo and De Jaegher (2015) with respect to theory of mind and the production of knowledge.

5 There is an important debate about the role played by direct picking up of information or mental processes as bases of perception. Gibson’s idea of perceiving excludes imagery and mental processes. On the other side, Neisser (1976, 1978), while trying to bring together ecological and cognitive psychology, develops the idea of perceptual cycle. This concept implies the fact that information picked up through perception activates what he calls *schemata*, which in turn guide attention and action leading to the search for additional information. Neisser (1978, p. 95) introduces the term anticipation, meaning: “What people see depends on the anticipations they develop, the perceptual explorations they carry out, and the information they find available; in other words, on the perceptual cycle in which they are engaged. Neisser’s idea of perceptional process is relevant in this context as it can be considered very close to our concept of processual landscape. Namely, what Neisser (1978, p. 92) calls “anticipation” in perceiving and in picking up information and affordances could be related to the cultural/social background belonging to the agent perceiving landscape (see also Milton, 2002, p. 42).

6 We are aware that there are many unsolved problems in addressing agency: one is surely its relationship with representational mental states, whether perception is accounted for by the relation between agent and environment or requires internal representations. Debate in philosophy of science and cognitive science shows that agency is possible even without representational mental states, both in human-being and generally in organisms (e.g., the notion of minimal agency and its adaptive character in Barandiaran et al. (2009; see also Moreno and Mossio, 2015, p. 89). A related issue concerns whether the agent perceives in a direct or mediated way, which is also an important topic in Gibson’s theory and its interpretations (Chawla and Heft, 2002, p. 206; Heft and Chawla, 2006, p. 20; Rietveld and Kiverstein, 2014). In addition, the thesis that our perception and esthetical judgment are agency-based has been recently supported by neurological literature and recent neuroimaging experiments (Di Dio et al., 2016): evaluation of artworks (depicting both human subjects and nature) involves the motor system as actively engaged by the depicted representations.
